# Sunstroke

**Published:** 1878-08

**Authors:** 


Sun Stroke.—We call attention to the treatment of sun stroke, or thermic fever, as it has been called by Dr. Wood. The antipyretic action of the cold bath and quinine makes these remedies exceedingly efficacious. The quinine should be given subcutaneously, as rapidity of action is necessary. One drop of dilute sulphuric acid and ten minims of water are necessary for the solution of one grain of quinine. The ordinary subcutaneous syringe will thus hold three grains.—*Ohio Medical Recorder.*

